# Development of a support system for measurement and analysis of thinking processes based on a metacognitive interpretation framework: a case study of dissolution of belief conflict thinking processes

**DOI:** 10.1186/s41039-018-0091-y

**Published:** 2018-12-17

**Authors:** Yuki Hayashi, Kazuhisa Seta, Mitsuru Ikeda

**Affiliations:** 1 Graduate School of Humanities and Sustainable System Sciences, Osaka Prefecture University, 1-1 Gakuen-cho Naka-ku Sakai-shi, Osaka, 599-8531 Japan; 2 Graduate School of Advanced Science and Technology, Japan Advanced Institute of Science and Technology, Nomi, Japan

**Keywords:** Metacognitive thinking processes, Gaze behaviors, Interpretation of thinking processes, Thinking task to dissolve belief conflicts

## Abstract

The ability for metacognitive thought, or “thinking about thinking,” is recognized as an increasingly important skill for the future enrichment of social life. However, this skill is difficult to teach because it involves implicit cognitive activities that cannot be perceived by an outside observer. In this study, we propose an interpretation framework of metacognition as one approach to considering metacognitive thinking processes. This framework serves as the design principles for developing a system that makes it possible to provide metacognitive interpretations of gaze behaviors and thought operation actions and provides a common basis for sharing and comparing knowledge from analysis results. In this study, for an example of framework-based system development, we construct a thinking externalization application and thinking analysis support system with a thinking task of dissolving belief conflict as the theme. We also demonstrate an example of the analysis of thinking about belief conflict, as derived from lower-level and higher-level thinking interpretation rules. From the example results of the defined interpretation rules, we found that the desired behavior occurred, demonstrating the postulated possibility of capturing the thought process. By realizing a series of phases on the framework proposed in this paper, it contributes to the feasibility of grasping the metacognition process and accumulating knowledge about it.

## Introduction

Owing to the multitude of beliefs in social life, there has been no shortage of attempts to directly confront difficult problems by defining unique solutions. In these cases, it is critical to cultivate metacognitive thinking abilities, the ability to “think about thinking.” Moreover, the ability to logically explain the thinking that takes place in our heads is increasingly recognized as an indispensable skill in social life (Livingston 1997; Griffin et al. 2012). Although such metacognitive skills are difficult to teach because of the implicit nature of thinking (Rana and Upton 2013; Kayashima and Inaba 2003), some studies have tackled the difficult task of developing this skill in the educational research fields. Veenman et al. claimed there are three types of fundamental principles for the acquisition and instruction of metacognition (Veenman et al. 2006): (a) embedding metacognitive instruction in the content matter to ensure connectivity, (b) informing learners about the usefulness of metacognitive activities to make them exert initial extra effort, and (c) prolonged training to guarantee the smooth and maintained application of metacognitive activity. On the basis of these principles, some studies on practical instructional programs have reported educational benefits in mathematical reasoning (Kramarski and Mevarech 2003) and business economics lessons (Masui and De Corte 1999). Pieger and Bannert investigated the effect of metacognitive prompts that inquire into the reasons for learning activities in order to foster learning behavior and learning outcomes in computer-based learning environments (Pieger and Bannert 2018). They compared three types of metacognitive prompt groups: a fixed prompt group in which students received fixed metacognitive prompts at fixed time intervals, a self-directed prompt group in which students received self-directed metacognitive prompts at self-chosen times, and control groups where students received no prompts. The results of learning outcomes revealed no differences between the three groups, but Pieger and Bannert found that learners’ verbal intelligence and reading competence affected the effectiveness of prompts. To diagnose learners’ metacognitive aspects, several measurement scales such as the metacognitive awareness inventory (Schraw and Dennison 1994) and metacognitive awareness of reading strategies inventory (Mokhtari and Reichard 2002) have also been used for analysis in several studies. However, research on metacognitive instruction often merely reports product measures (i.e., the effects on learning outcomes). To establish causal relationships between metacognitive instruction and learning outcomes, process measures of metacognition also need to be assessed in a pre-post-test design, and the comprehension of the complex nature of metacognitive processes, which is an essential component of successful self-regulation, has become an important topic (Azevedo 2009; Azevedo et al. 2009; Veenman et al. 2006).

To capture and provide meaningful data during the implicit thinking process, the “thinking-aloud method,” wherein one completes a task while successively vocalizing everything one is thinking, has been utilized (Jaspers et al. 2004; Azevedo et al. 2007). Although this method is commonly used for analyzing the thinking process, several problems with it have been identified: it is difficult to collect data on subjects in their natural state; completing a task while verbalizing each step raises cognitive load; and task executioners with low introspective abilities find it difficult (Miyazaki and Miyazaki 2004; Jääskeläinen 2010). Alternative techniques have also been documented, such as the retrospective method of answering questions about one’s thinking during a task after it is completed, or stimulated recall in which one watches and reflects on a video recording of the task (Fox-Turnbull 2009); however, these require introspective work by the thinking subject.

In response to these problems, this study focuses on one’s “gaze behavior” during the thinking process. In general, eyeball movement occurs as a repetitive alternation between a saccade, as one shifts the focus of one’s gaze when trying to see an object, and a stationary gaze at the object (Barlow 1952). There are four broad categories of eye movement measurement methodologies: electrooculography (EOG), scleral contact lens/search coil, photo-oculography (POG) or video-oculography (VOG), and video-based combined pupil and corneal reflection. In these methods, video-based trackers utilize relatively inexpensive cameras and image processing hardware, so that they are most suitable for use in interactive systems (Duchowski 2007). Because the gaze is affected by the thinking process, studies have been conducted in cognitive psychology that use physiological gaze data as a clue to approach thinking, e.g., variations in the gaze during the verbalization (Guan et al. 2006) and reading comprehension processes (Reichle et al. 2010). Along with the development and cost reduction of a measurement device for gaze behaviors, eye tracking technologies has been widely used in not only cognitive psychology but also marketing and advertising, human–computer interaction, and collaborative systems.

In the research field of educational technology, several studies have attempted to understand how gaze information can be used and applied to diagnose, explore the cognitive processes, or support learning over the last decade. For instance, Lai et al. reviewed empirical studies that have employed the eye tracking technology to probe the cognitive processes during learning (Lai et al. 2013). They analyzed 113 eye tracking studies from 2000 to 2012 based on the social sciences citation index database. They found that the number of eye movement studies related to learning has increased significantly since 2009. The result of content analysis showed that eye movements and learning were studied under the following seven themes: patterns of information processing, effects of learning strategies, reexaminations of existing theories, individual differences, effects of learning strategies, patterns of decision making, and social cultural effects. They also found that many of the studies used quantitative temporal and count data widely for the learning themes, while spatial indicators (e.g., fixation location and scan path) were the least used ones. In the same vein, Yang et al. also reviewed 33 e-learning related eye tracking studies from 2005 to 2014 that were selected from the Social Science Citation Index database (Yang et al. 2018). They found that most of the studies analyzed eye movement measures comparatively with achievement test, skill/ability test, and performance exam to explain consequent learning behaviors or performances. Based on the clustering analysis of the research methods and findings, they provided instructional suggestions for supporting science learning in digital environments, such as placing related text and graphics near each other and employing only one type of verbal mode of information.

As examples of more concrete studies in the field of intelligent tutoring systems, Bondareva et al. explored the value of gaze data in diagnosing student learning during interactions with MetaTutor, which is an adaptive hypermedia learning environment to scaffold students’ self-regulated learning (Bondareva et al. 2013). They use two types of gaze features: summary statistics of general gaze trends such as the mean and standard deviation of fixations and saccade length, and statistical gaze features of a MetaTutor interface-specific area of interest (AOI) such as text-content and image-content area. They adapted several types of classification algorithms to predict student learning (the learning performance of high- or low-level learners measured by pre- and post-tests) and achieved 78% classification accuracy on the basis of gaze data. There are also some studies to estimate the learners’ internal states during learning task execution. Jaques et al. investigated the usefulness of gaze data for predicting students’ self-reported emotions (boredom and curiosity) during interactions with MetaTutor (Jaques et al. 2014). To do this, they used several types of application-dependent/independent statistical gaze features as well as Bondareva et al.’s analysis. They analyzed four types of classification algorithms such as Logistic Regression and Support Vector Machines applied to the gaze features computed using various time intervals, and the best results were 69% classification accuracy for boredom and 73% for curiosity. Lallé et al. investigated occurrences of learners’ self-reported confusion during the interaction with ValueChart, which is an interactive visualization to support multi-criteria preferential choice (Lallé et al. 2016). For the analysis, they used the statistical amount of eye tracking data of general/AOI-dependent gaze and pupil features, head distance features from the screen, and mouse clicking features on the interface. They built classifiers using the random forest algorithm to classify learners’ confusion/non-confusion, and successfully predicted 61% of learners’ confusion by training all the above features. However, most of these studies tend to analyze not the process but quantitative trends in learners’ gaze measurement results.

As a methodology to analyze a user’s cognitive process on the basis of his/her eye movement information, Salvucci and Anderson proposed a class of tracing methods (target tracing, fixation tracing, and point tracing) for automatically analyzing eye movement protocols on the basis of the Hidden Markov Model (Salvucci and Anderson 1998; Salvucci and Anderson 2001). The methods require three types of inputs: a sequence of eye movement data by an eye tracker, a set of target AOIs, and a process model described as regular grammar (Sudkamp 1988) that provides tracing with a specification of the set of possible predicted action sequences. The methods estimate which process was executed by matching a sequence of eye movement data with the process model. They were also validated in several task domains, such as the work of solving an equation, reading comprehension, and eye-typing, in which users type by looking at characters on an on-screen keypad. The results provided a clear picture of how the tracing methods benefit different types of applications (Salvucci and Anderson 2001). Although this methodology is a general-purpose one for analyzing a user’s gaze process, no methodological framework exists for interpreting the implicit metacognitive thinking processes targeted at base-level thinking processes, as far as we know.

Metacognitive monitoring and control is performed on base-level thinking during internal self-conversation (National Research Council 2000) occurring inside the head. In context-independent conditions, it is of course difficult to capture the entire contents of a person’s metacognitive thinking from gaze data alone. Assuming a thinking externalization application that has an interface that establishes a correspondence between a specific thinking task context and its outcomes (without constraints of a range that would create a sense of discomfort during use), we hypothesize that a part of the metacognition process that is indefinite and strongly individually determined can be grasped from not only the acts of reviewing and correcting externalized thinking (hereinafter, *thought operation actions*) but also a person’s *gaze behaviors* while he/she is engaged in a task.

In this study, we elaborate on a model whereby premises to grasp the formless process of thoughts taking place in the head are explicitly defined to interpret thinking processes. On the basis of this model, we propose an interpretation framework for metacognition that could serve as a common foundation for system development. Our framework provides a structure for the work process necessary to capture the thought process and defines the system of concepts necessary to carry out these tasks. Furthermore, to demonstrate that our proposed framework can take our idea of capturing metacognition through to the development of a support system to measure and analyze actual metacognitive processes, we develop a metacognition process measurement system (thinking externalization application) and analysis support system on the basis of the framework. To demonstrate that a mechanism leading to system development and utilization can be realized, we present the results of a case study of metacognitive interpretation in a corrective context using a thinking task of dissolving belief conflict as an example. The contributions of this study are twofold:

1. We propose an “interpretation framework” for metacognition that could serve as the design principles for developing a system that makes it possible to provide metacognitive interpretations on the basis of gaze behaviors and thought operation actions (“Metacognitive interpretation framework for understanding metacognitive processes”, “Base-level thinking representation ontology”, and “Notation for interpretation rule definition” sections); and
2. We demonstrate the “feasibility” of specific work processes of the proposed framework by designing, developing, and utilizing a thinking externalization application and thinking analysis support system around the theme of a thinking task to dissolve belief conflict (“Example system development based on framework” and “Interpretation of thinking processes based on interpretation rules” sections).

In “Example system development based on framework” section, we first clarify the target metacognitive processes. Then, we explain the concept behind the interpretation framework of metacognitive thinking and how it can be used to understand the thinking process, as well as the operative details for stakeholders intending to use the framework to analyze the metacognitive process. In “Interpretation of thinking processes based on interpretation rules” section, we explain the base-level thinking representation ontology that plays a role as an explicit specification connecting the concept of this framework to the development of a thinking analysis system. With reference to this ontology, “Interpretation of thinking processes based on interpretation rules” section explains the notation to define interpretation rules that provide interpretations able to capture base-level and meta-level thinking. The “Example system development based on framework” section demonstrates both a thinking analysis support system and a thinking externalization application for the thinking task of dissolving belief conflict as real examples of system development based on the interpretation framework. Finally, “Interpretation of thinking processes based on interpretation rules” section illustrates these examples with applied results of interpretation rules in the thinking analysis support system.

## Metacognitive interpretation framework for understanding metacognitive processes

### Target metacognitive processes

Since the term “metacognition” is very often used in educational literature in various learning domains and is sometimes used interchangeably, its definition is often a topic of discussion (Dinsmore et al. 2008; Lajoie 2008; Livingston 1997). In general, metacognition consists of both metacognitive knowledge and metacognitive experiences or regulation (metacognitive activities), where metacognitive knowledge refers to acquired knowledge about cognitive processes that can be used to control cognitive processes (Flavell 1987). The basic and most widely known model of metacognitive activities consists of three principles: mental processes are split into two or more specifically inter-related levels, a *cognitive* and a *metacognitive* level; the metacognitive level contains a dynamic model of the cognitive level; and there are two dominance relations called control (*metacognitive control*) and monitoring (*metacognitive monitoring*), which are defined in terms of the direction of the information flow between the *meta*-*level* and the *object*-*level* (*base*-*level*, in this paper) (Nelson and Narens 1994). Examples of the metacognitive monitoring include being aware of, feeling, predicting, checking, and evaluating cognition, and those of metacognitive control include setting goals for planning and modifying cognition.

This study especially focuses on the situation in which the thinker demonstrates metacognition about what externalized (written) product of one’s/other’s thoughts at the base-level is needed to verify their logical structure. In this situation, the thinker needs to perform metacognition (e.g., checking, comparing, evaluating, and modifying) for what he/she is trying to write (cognition) inside his/her head. Therefore, we take the standpoint of Hacker et al.’s metacognition model, which expands the Nelson and Narens’s model (Nelson and Narens 1994) as applied to writing (Hacker et al. 2009), even though there are some distinctions between definitions. In this model, control information flows from the meta-level to the base-level to modify the state of the base-level process or knowledge or to change the base-level altogether. Modifications or changes at the base-level are monitored at the meta-level so that the base-level model can be modified. A change in the state of the base-level model can lead to additional control information flowing to the base-level (Hacker et al. 2009).

### Interpretation model for metacognitive processes

**Fig. 1:**
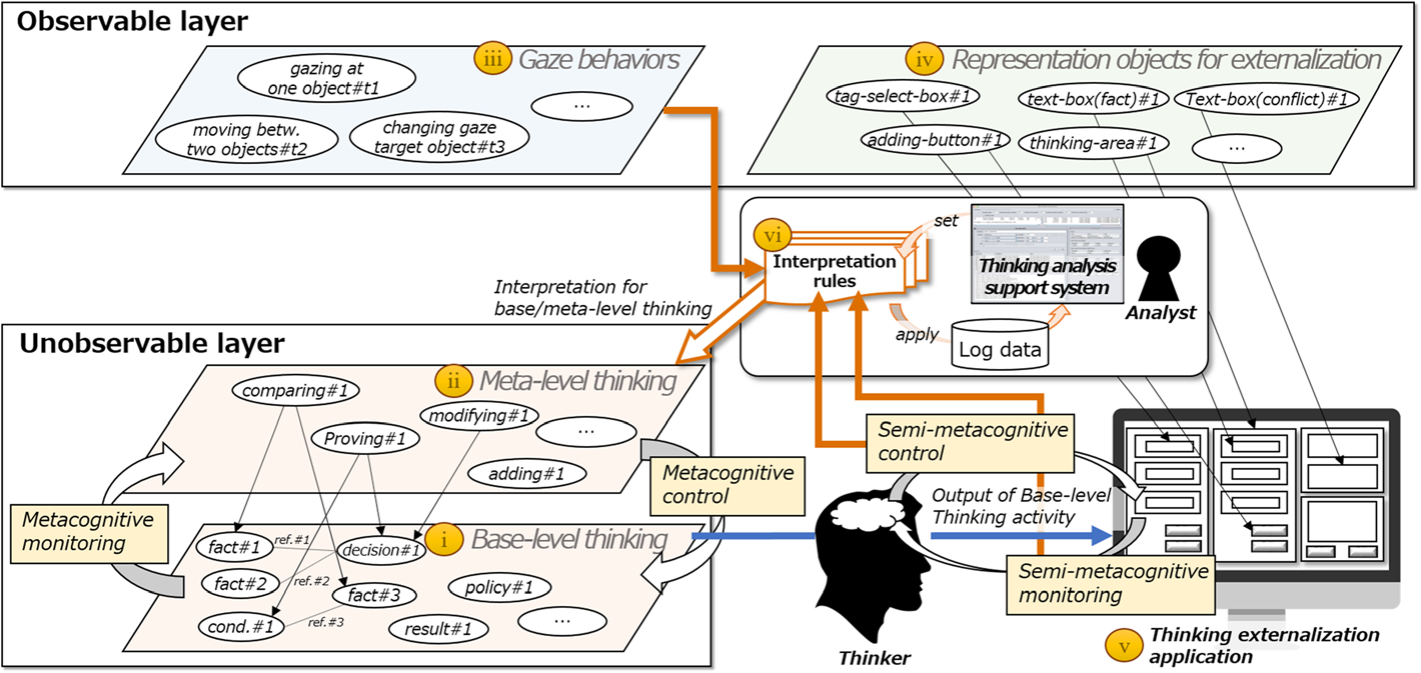
Interpretation model to capture meta-level thinking processes

Figure 1 shows the premises of this study that enable meta-level thinking processes to be captured in a schematization of the interpretation model of the metacognitive thinking process. The thought process, which takes place in an area that cannot be observed from the outside world, is understood to be composed of two layers (Nelson 1990; Hacker et al. 2009): “base-level thinking” (Fig. 1(i)) and “meta-level thinking” (Fig. 1(ii)), where the meta-level thinking monitors (metacognitive monitoring) and controls (metacognitive control) the base-level thinking.

Gaze behaviors can be measured from the outside world by using eye trackers and other gaze measurement devices (Fig. 1(iii)). By specifying gaze times, gaze behaviors such as “gazing at an object,” and “change in target object of gaze” can be tracked from a series of information about saccades and fixations.

Our basic idea is to assume that the logical relationships between the products of base-level thinking activity taking place inside the head can be reflected and externalized into an interface, thus allowing meta-level thinking performed by the thinker to be captured via gaze behaviors and thought operation actions. For example, in the thinking activity, “decide where to go tomorrow,” where one might “take as a ‘*basis the fact that the chance of rain is 80%*,’ ‘*hypothesize that it will rain*,’ and ‘*decide to go to the aquarium*,’” the products of thought are created in the head. However, we propose they could be externalized onto an interface. In this study, we refer to the results of such thinking activity as the “output of base-level thinking activity.” In addition, we introduce the concept of “thinking representation objects for thought externalization” to externalize the output of base-level thinking activity in a visible form (Fig. 1(iv)). These representation objects for thought externalization correspond with commonly used components in graphical user interface (GUI) applications such as “panel,” “text-box,” and “selection-box.” By assuming that the output of base-level thinking activity can be externalized onto these representation objects, it becomes possible to observe the gaze behavior and thought operation acting on it.

In this study, we focus on the isomorphism between the “metacognitive monitoring and control of base-level thinking taking place inside the head” and “gaze behaviors and thought operation actions related to the output of base-level thinking externalized to thinking representation objects.” By assuming the thinking externalization application where one can externalize base-level thinking results as the output on appropriate representation objects (Fig. 1(v)), we hypothesize that a part of the meta-level thinking process might be captured from gaze behaviors and thought operation actions. Here, we consider gaze behaviors and thought operation actions related to the output of base-level thinking as “semi-metacognitive monitoring and control” activity.

The structure of the thinking task influences the way that metacognition monitors and controls base-level thinking. For example, if a teacher evaluates learners’ written documents/reports in terms of learners’ bug detection/correction, the teacher is asked to engage in thinking activities such as understanding (metacognitive monitoring) and modifying (metacognitive control) the described base-level thought of others (learners). To cope with the diversity of interpretations of metacognition influenced by the structure of the thinking task, we introduce “interpretation rules” (Fig. 1(vi)). Interpretation rules are formally represented rules that extract sections of base-level and meta-level thinking from the gaze behaviors and thought operation actions, including the activities of semi-metacognitive monitoring/control, and interpret them. These interpretation rules are assumed to be defined and applied by an analyst through the thinking analysis support system.

In the framework we propose, we systematize an ontology (base-level thinking representation ontology) to formally express the output of base-level thinking activities related to the task and externalized thought components as representation objects for thought externalization. Because it clarifies which gaze behaviors and thought operation actions in response to representation objects for thought externalization should be understood as which semi-metacognitive monitoring/control activities, using this ontology as a foundation makes it possible to implement the thinking externalization application so as to clarify the application’s design principle. Furthermore, by defining interpretation rules on the basis of the concepts of this ontology, analysts’ intentions can be clarified within the series of processes from measurement for interpretation of metacognition to interpretation itself in a form that facilitates agreement about the results.

### Metacognitive interpretation framework

Our meta-level thinking process interpretation framework is specified in Table 1 on the basis of the interpretation model shown in Fig. 1. Our framework gives a working process to capture a thinker’s thinking process, and we specify both concepts necessary for carrying out this work: the base-level thinking representation ontology (Table 1(1) and (2)) and the notation to specify interpretation rules (Table 1(3)). This section describes the working process (Table 1, bottom row), and “Base-level thinking representation ontology” and “Notation for interpretation rule definition” sections describe the concepts specified by the framework (Table 1, top row) in detail.

**Table 1:** Interpretation framework to capture meta-level thinking processes

	Framework concepts		Contents	Operation reference
(1)	Base-level thinking representation ontology	Output concepts of base-level thinking activities	Ontology that specifies the output concepts of base-level thinking activities in a target thinking task (“Output concepts of base-level thinking activities” section)	(A), (B), (C), (E)
(2)		Concepts of thinking representation objects	Ontology that specifies thinking representation objects as parts for thought externalization (“Concepts of representation objects for thought externalization” section)	(B), (C), (E)
(3)	Notation to specify interpretation rules		Notation for interpreting captured base-level and meta-level thinking from a series of gaze behaviors and thought operation actions (“Notation for interpretation rule definition” section)	(C), (E)
	Phase	Stakeholders	Work	Contents
(A)	Design	Analyst	Realize (1)	Add thinking-task-specific output concepts by extending an ontology of (1).
(B)	Development	Analyst and developer	Map and realize (1) → (2)	Define correspondence among the base-level thinking concepts specified in (A) and the representation objects for thought externalization, and develop a thinking externalization application on the basis of it (“Eye-Sizhi” section)
(C)		Developer	Realize (3) on basis of (1) and (2)	Define interpretations of gaze behaviors and thought operation actions measured by thinking externalization application of (B) as instances of interpretation representation classes, and develop a thinking analysis support system to capture semi-metacognitive monitoring and control activities (“Thinking analysis support system” section)
(D)	Measurement	Thinker	–	Using thinking externalization application of (B), perform thinking task (gaze behaviors and thought operation actions are measured.) (“Target data” section)
(E)	Analysis	Analyst	Apply (3)	Using the thought analysis support system of (C), specify interpretation rules of gaze behaviors and thought operation actions measured in (D), and analyze the results (“Applied results of interpretation rules” section)

Specific work processes are organized into four phases: design, development, measurement, and analysis. We assume the existence of someone to analyze the thinker’s metacognitive thinking (analyst), someone to develop the system used in analysis (developer), and someone whose thinking is the subject of analysis (thinker) as stakeholders corresponding to each task.

Design phase (A): The analyst specifies analytic thinking-task-specific concepts by extending an ontology in which the output concepts of base-level thinking activities are defined.

Development phase (B): Correspondences among the output concept of the base-level thinking activities specified in (A), and the representation objects for thought externalization specified in advance in an ontology (2), are discussed and defined by the analyst and developer. They develop a thinking externalization application that adopts an interface composed of instances of the thinking representation objects for thought externalization on the basis of these correspondences.

Development phase (C): A thinking analysis support system is developed that can extract the subjects of analysis from gaze behaviors and thought operation actions measured in (B) and where interpretation rules (“Interpretation of thinking processes based on interpretation rules” section) can be defined.

Measurement phase (D): The thinker performs the thinking task through the thinking externalization application constructed in (B).

Analysis phase (E): Interpretation rules are specified and deployed on the thinking analysis support system developed in (C) and applied to the thinker’s gaze behaviors and thought operation actions measured in (D), and through iterative analysis, a verifiable hypothesis on the thinker’s metacognitive processes is captured.

In general, metacognition itself is a vague concept, so that it is impossible to clearly distinguish cognition from metacognition inside the head and capture such processes from the outside world under normal conditions. Furthermore, the vagueness of the concept is the main reason which makes it hard to accumulate knowledge about system to support metacognition; without clarifying what kinds of metacognition each system tries to support, it is conceivable that systems’ support target might be quite different, even though the same term of “metacognition” is used to designate their focus. To overcome this difficulty, the proposed framework explicitly requires the work process to specify the premise concepts of target cognition (base-level thinking) that are assumed on the target thinking task as an ontology (output concept of base-level thinking activities) in (A). Henceforth, the design intention behind the thinking externalization application can be stated in the following terms: on the basis of the specified concepts resulting from limiting the target thinking task, it becomes possible to make clear what type of cognition (base-level thinking) are externalized on the interface in (B). This is a promising idea that increases the chance of capturing a part of meta-level thinking process since a thinker use the application by monitoring (gaze behaviors) and correcting externalized base-level thinking (thought operation actions) in (D). More generally, the target content-dependent cognition (base-level thinking) can only be clearly revealed by limiting (specifying) the target thinking task, whereas generality can only be achieved without limiting the target thinking task; it is a trade-off problem. In our study, we take the former approach to make it possible to develop a thinking externalization application that explicitly externalized the premise of the target cognition in the proposed framework. As a result, we can demonstrate the connectivity between metacognitive monitoring and control for task-dependent base-level thinking on the one hand, and gaze behaviors to thought operation actions on the other hand, as will be described in this paper.

We explain the base-level thinking representation ontology defined in the framework in “Base-level thinking representation ontology” section, and the notation to specify interpretation rules in “Interpretation of thinking processes based on interpretation rules” section.

## Base-level thinking representation ontology

The base-level thinking representation ontology (Table 1(1) and (2)) defined in the framework forms the basis for maintaining the continuity from the interpretation model of the metacognition processes, shown in Fig. 1, to the development of the thinking externalization application and thinking analysis support system. Here, concepts shared independently of any thinking task types are defined, and the analyst defines the output concepts of base-level thinking activities specific to the target thinking task (Table 1(A)) under the shared concepts so that the basis for system development can be established.

### Output concepts of base-level thinking activities

**Fig. 2:**
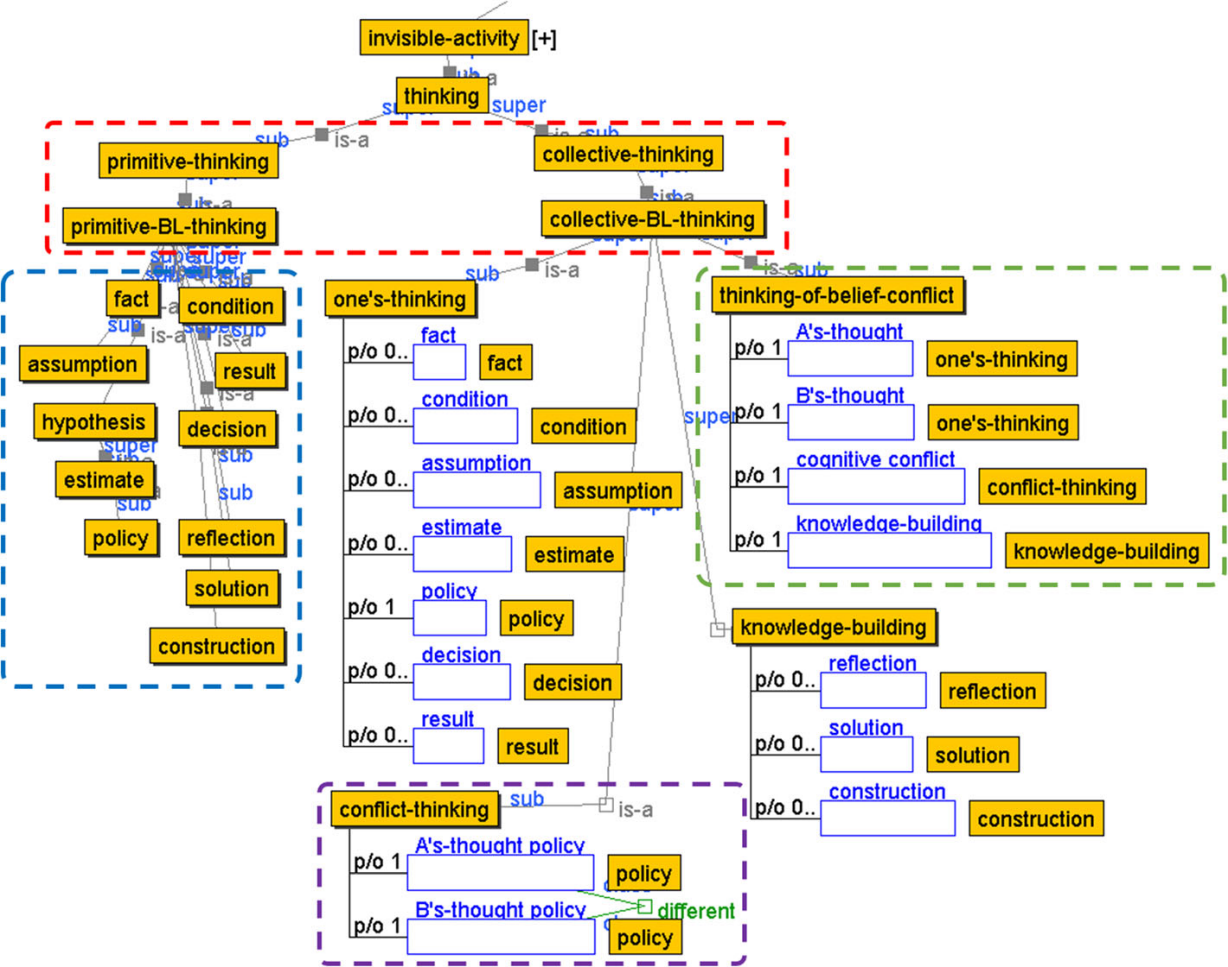
Concepts of output of base-level thinking activity in thinking of belief conflict

Figure 2 shows the output concepts of base-level thinking activity (hereafter, “base-level thinking concepts”) in the thinking representation ontology. We use an ontology building environment called Hozo (http://www.hozo.jp/hozo/). Hereafter, initial appearances of certain concepts are underlined in the text. Even though we can say “thinking,” it can be captured in a variety of ways in accordance with the target thinking task types. To specify differences in the granularity of such thinking activity, the output concepts of base-level thinking activity need to be categorized into two major concepts: the primitive concept (primitive-BL-thinking) representing the minimum unit of base-level thinking activities under the thinking concept (thinking), and the base-level collective concept (collective-BL-thinking) that expresses the integrated whole (Fig. 2, red frame).

Primitive concepts are specified as subclasses of primitive-BL-thinking of the target thinking task. In the example of a thinking activity in thinking of belief conflict, “fact,” “condition,” “estimate,” “policy,” “decision,” “result,” etc., which are specified on the basis of the ontological concept proposed by Chen et al. 2011, correspond to the primitive concepts (Fig. 2, blue frame). We allow analysts to specify additional concepts (phase (A) in Table 1). For example, if an analyst refers to the idea of Toulmin’s argument method (Toulmin 2003), he/she can add the necessary primitive concepts such as “argument,” “claim,” “warrant,” and “backing.”

For the subclass concepts of collective-BL-thinking, the analyst specifies the thinking concepts in accordance with the target thinking task as it is composed of an aggregate of primitive-BL-thinking (Table 1(A)). In Fig. 2, we describe a system of concepts as an example of a “thinking task of dissolving belief conflict” (thinking-of-belief-conflict) (“Target thinking process and its training program” section), which this paper takes as its case study. Thinking-of-belief-conflict is defined to be composed of (part of) the concepts of one’s own thinking and another’s thinking (A’s-thought, B’s-thought) representing the logical paths of base-level thinking that cause belief conflict, the conflict concept representing the origin of the conflict for each thought (conflict-thinking), and the concept of knowledge building to overcome the conflict (knowledge building) (Fig. 2, green frame). Furthermore, the concepts of one’s-thinking, conflict-thinking, and knowledge building are defined as subclasses of primitive-BL-thinking. Conflict-thinking, for example, represents the root of belief conflict and is defined as a conflict between two different judgment policies of A’s-thought and B’s-thought (Fig. 2, purple frame). In design stage (A), analysts specify the base-level thinking concepts in the target thinking task as collective-BL-thinking, which is composed of primitive-BL-thinking.

Primitive- and collective-BL-thinking are defined as invisible-activity that cannot be observed from the outside world. In the meta-level thinking which targets those base level thinking processes, thoughts are performed such as “to think about the premise of a given judgment,” “to compare policies leading to a belief conflict,” “to examine whether an assumption is valid,” and “to check and adopt different judgment policies.” Additionally, the framework provides the notation to define interpretation rules (“Notation for interpretation rule definition” section) for capturing such kinds of meta-level thinking dependent on these thinking tasks.

### Concepts of representation objects for thought externalization

As described in the “Introduction” section, this research takes as a premise the interface (thinking externalization application) that establishes a correspondence between the thinking task context and the thought results, and the respective outputs of base-level thinking activities introduced in the previous section are verbalized into representation objects for thought externalization. The framework defines this premise as the relationship between the base-level thinking concept and the representation objects for the thought externalization concept.

**Fig. 3:**
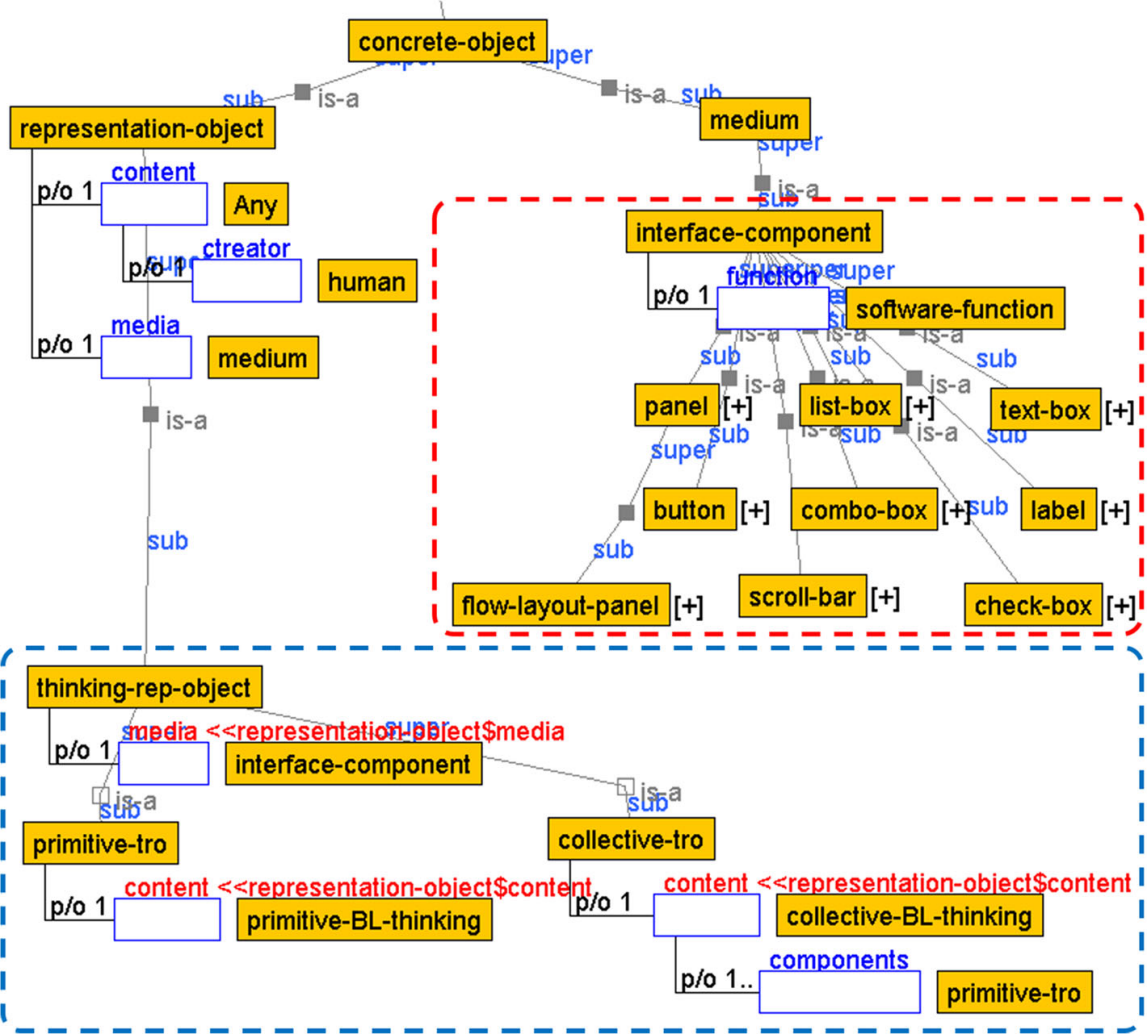
Concepts of thinking representation objects

Figure 3 shows an ontology that organizes the representation objects for thought externalization. We define the medium and representation object as subclass concepts of a concrete-object, which is composed of content and media.

nterface components such as panels, buttons, labels, and text-boxes are defined as subclass concepts of the interface component, a concrete example of medium, and are specialized in accordance with individual software functions (software-function) (Fig. 3, red frame).

As the subclass concept of representation object, thinking representation object (thinking-rep-object) for thought externalization is specified as the concept composed of specialized interface components (Fig. 3, blue frame). To associate the externalized thoughts with the base-level thinking concepts described in “Output concepts of base-level thinking activities” section, the content constituting the primitive thought representation object concept (primitive-tro) is specialized that stores primitive-BL-thinking, and the content constituting the collective thought representation concept (collective-tro) is specialized that stores collective-BL-thinking. Thus, the collective-tro is defined as an aggregate of the primitive-tro. Therefore, it represents the design intention of the thought externalization application about what types of base-level thinking concepts (primitive- and collective-BL-thinking) are verbalized on what kinds of representation objects for thought externalization (primitive- and collective-tro).

## Notation for interpretation rule definition

By constructing the thinking externalization application on the basis of the base-level thinking representation ontology in the previous section, the gaze behavior and thought operation actions of the user working on the thinking task can be measured in association with the thinking concepts defined in the ontology. In this section, we describe the premise of the interpretation process to the measured raw data, as well as the notation to specify interpretation rules (Table 1(3)), on the basis of this premise.

**Fig. 4:**
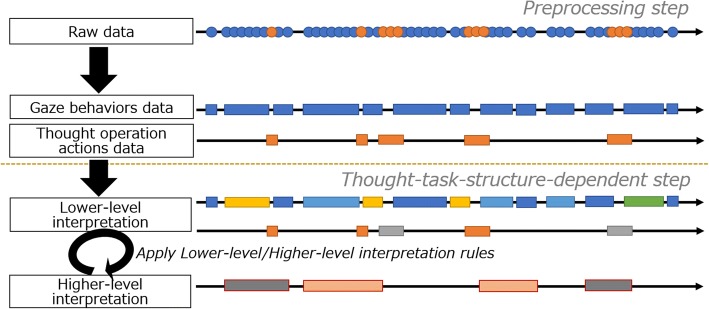
Concept image of applying interpretation rules

Figure 4 shows a conceptual diagram to apply interpretation rules. Interpretation rules that capture the characteristics of the thinking task structure are applied (Fig. 4, lower part) through a preprocessing stage that is independent of the thinking task structure (Fig. 4, upper part).

### Preprocessing

This process involves refining raw data consisting of gaze behaviors and thought operation actions for thinking representation objects. It is realized by eliminating noise on the basis of a predefined gaze fixation time. Moreover, by setting a time interval, it is determined whether a series of gaze behaviors involving gazing at one thinking representation object with very short breaks and thought operation actions can be recognized as one act or not.

### Thinking task structure-dependent processing

This process involves interpreting each data series classified via preprocessing. Interpretation processing consists of two steps: application of a lower-level interpretation rule that provides a lower-level interpretation (lower-level interpretation); and for sections given an interpretation in this stage of processing, application of a higher-level interpretation rule (higher-level interpretation) that gives interpretations including meta-level thinking processes.

### Notation for lower-level interpretation rules

The notation to define an interpretation rule that provides the lower-level interpretation *PI*_*L* to the data series of gaze behaviors and thought operation actions is expressed by (1):

1 $$ {lr}_x\left( Act\left({tro}_i\right), PI\_L\right). $$

The first argument *Act*(*tro*_*i*_) represents a data series of gaze behaviors and/or thought operation actions on the thinking representation object (*tro*_*i*_) in the above preprocessing. Elements of gaze behaviors include *GazeAt*(*tro*_*i*_), and elements of thought operation actions include *Keypress*(*tro*_*i*_), *Delete*(*tro*_*i*_), and *Press*(*tro*_*i*_) and so on. The expression shows that the lower-level interpretation *PI*_*L* of the second argument is given to the time interval identified here.

### Notation for higher-level interpretation rules

The notation to define the interpretation rule that provides the higher-level interpretation *HI*_*L*, i.e., base-level thinking and the metacognition level interpretation, to the series of lower-level interpretations is shown in (2):

2 $$ {hr}_x\left( Cond, HI\_L\right). $$

*hr*_*x*_ represents the function that detects the matching intervals that satisfy the condition *Cond* and gives a higher-level interpretation *HI*_*L* of the intervals. The first argument *Cond* consists of one or more sets of conditional function(s). Table 2 summarizes the conditional functions and their explanations that are used to set *Cond* on the basis of the possible relationship between two intervals. At present, we select the functions available for time series analysis with reference to Allen’s time interval logic (Allen 1983). As shown in Table 2, arguments *L*_1_ and *L*_2_ represent the interpretation results of data intervals deduced by the lower-level/higher-level interpretation rules. *All* (*L*_1_) represents all data of *L*_1_. *BEFORE* (*L*_1_, *L*_2_, *t*) expresses the data section in which *L*_1_ appears *t* ms before *L*_2_. *OVERLAPS* (*L*_1_, *L*_2_) detects the overlapped intervals between *L*_1_ and *L*_2_, and *DURING* (*L*_1_, *L*_2_) extracts the intervals *L*_1_ if the time duration of *L*_2_ includes the one of *L*_1_.

**Table 2:** Function list for condition setting

Function	Interpretation
*ALL* (*L*_1_)	*all L*_1_
*BEFORE* (*L*_1_, *L*_2_, *t*)	*L*_1_ *takes place before L*_2_ *within* t *ms.*
*OVERLAPS* (*L*_1_, *L*_2_)	*L*_1_ *overlaps with L*_2_
*DURING* (*L*_1_, *L*_2_)	*L*_1_ *during L*_2_

Applications and other specific examples of lower- and higher-level interpretation rules are described in “Applied results of interpretation rules” section.

## Example system development based on framework

This section shows an implementation example of the thinking externalization application and thinking analysis support system based on our framework. This work corresponds to the implementation of the design and development phases (A) to (C) in Table 1, and in this system development, the analyst and system developer are the authors. In “Target thinking process and its training program” section, we explain the thinking process of “task of dissolving belief conflict” that we focus on in this study, and outline the “Sizhi” training environment. The “Eye-Sizhi” section takes a thinking externalization application called “Eye-Sizhi” developed on the basis of the Sizhi, which is equipped with a training environment for thinking to dissolve belief conflicts based on our framework. The “Thinking analysis support system” section establishes interpretation rule settings for interpreting data series of gaze behaviors and thought operation actions measured by Eye-Sizhi and explains the thinking analysis support system for visualizing the interpretations on the basis of the rules.

### Target thinking process and its training program

Ito has examined the verbalization effect of thought from the viewpoint of learning strategies and proposed a goal-attainment model as a learning strategy composed of three processes: the knowledge description cycle, clarify cognitive conflict point, and the knowledge building cycle (Ito 2009). According to this process, it is assumed that by actively conducting an internal self-conversation focused on thinking logically, one’s own thoughts are refined, and the thinking process itself is clarified (Seta et al. 2013).

On the basis of this goal-attainment model, the learning environment “Sizhi” to promote internal self-conversation has been proposed to train the “thinking about thinking (meta-level thinking)” skill (Chen et al. 2011). When building sophisticated knowledge, the essential causes of conflicting beliefs in different value systems need to be dug up. In Sizhi, the subject is to construct knowledge for dissolving belief conflict structures, which are based on one’s own experience and have no correct answers (Kyougoku 2011; Kyougoku 2015), and to verbalize the logical paths of one’s own judgments (A’s-thought) and other’s judgments (B’s-thought) in terms of a series of “statements (the smallest units of thinking)”. Next, after specifying “policies” that form the beliefs of A’s-thought and B’s-thought and clarifying those “evidences,” “conflict” as the root cause responsible for those belief conflicts is clearly expressed. In addition, a solution (knowledge building) is constructed to overcome it. To clarify and increase awareness of the logical structure of one’s own thinking, thinking activities are necessary to consciously and explicitly give each statement a “Sizhi tag,” which represents the role it plays in the logical structure.

Sizhi has been implemented in classes for first-year university students (Seta et al. 2013) as well as for medical care workers in a knowledge building method workshop (Kanou et al. 2013), with improvements in learners’ metacognitive skills being reported.

However, because the thinking process leading to expressions of thought on Sizhi remains implicit, its interpretation is left to the learner who reflects on his/her own thought or the corrector who reviews or corrects it. If there is a possibility of interpreting this implicit thinking process, we believe that we can capture a part of the thought process of learners and correctors that could not be easily grasped before. This is despite the fact that it has been to date impossible to understand both the differences observed between learners in thinking expression processes that clarify root causes of conflicts and the differences in the corrections of thinking process between those experts with abundant experience of correction and correctors with relatively little experience.

### Eye-Sizhi

**Fig. 5:**
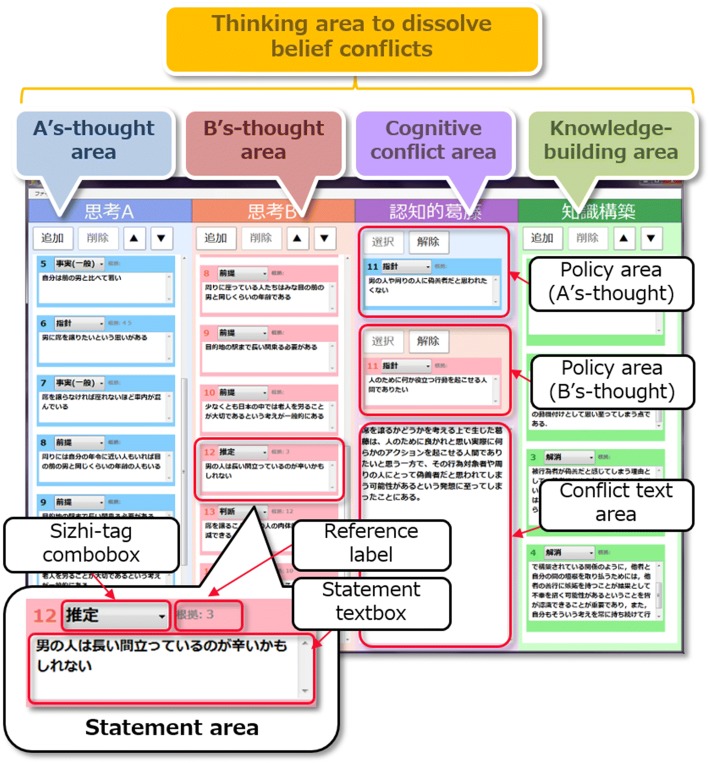
Eye-Sizhi interface

For a learning support tool to encourage verbalization of thinking to dissolve belief conflicts, we built an application called “Eye-Sizhi” that can measure users’ gaze behaviors and thought operation actions on the basis of our framework. Figure 5 depicts the Eye-Sizhi interface. Implemented in a form that follows from the Sizhi design philosophy, it allows for verbalization of thinking to dissolve belief conflict structures in four thinking areas: “A’s-thought,” “B’s-thought,” “cognitive conflict,” and “knowledge building,” all of which are included in the thinking area to dissolve belief conflicts. This application is based on the base-level thinking representation ontology described in “Base-level thinking representation ontology” section.

Specifically, in framework tasks (A) and (B) listed in Table 1, the base-level thinking concepts primitive-BL-thinking, and collective-BL-thinking, focused on the thinking task of dissolving belief conflict shown in Fig. 2. They are defined as contents of verbalization corresponding respectively to the statement area in thinking representation objects (Fig. 3) and the thinking area concepts. Then, on the basis of these specifications, Eye-Sizhi is developed in (C). By developing the thinking externalization application in this way, the representation objects on the application interface clarifies what type of output of base-level thinking activity (cognitive activity) are externalized. This is the reason why the thinker’s interaction on the thinking representation objects (gaze behaviors and thought operation actions) increases the chance of capturing a part of meta-level thinking process (i.e., semi-metacognitive monitoring and control processes).

Using a stationary eye tracker, Eye-Sizhi has the ability to track the objects at which users are gazing. Each thinking representation object for thought externalization located on the interface is given an area of interest (AOI), and the system records information in millisecond units about the point in time at which a user’s gaze coordinates on the screen entered or exited an object’s AOI. Target objects for which an AOI is set include thought areas (A’s-thought, B’s-thought, conflict, knowledge building), statement areas, and conflict text area. In addition to automatically detected gaze behavior information, keyboard input operations on thinking representation objects and operation of the mouse are recorded as thought operation actions.

### Thinking analysis support system

**Fig. 6:**
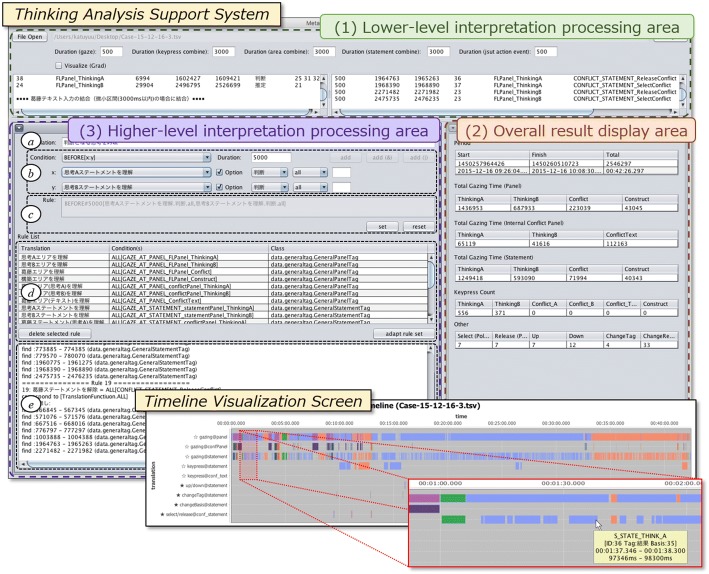
Thinking analysis support system interface

We developed a thinking analysis support system that takes an Eye-Sizhi log file as input, applies the lower-level and higher-level interpretation rules described in “Notation for interpretation rule definition” section, and visualizes the results. Figure 6 shows the system interface. The system is largely composed of the three areas outlined below, and a timeline visualization screen of interpretation results.

1. Lower-level interpretation processing area: In this area, the analysts can set three types of time intervals on the millisecond time scale: (1) gaze time; (2) coupling interval time of the gaze behaviors and thought operation actions that occur together (gazing at the thinking areas, gazing at the statement areas, keyboard input operations); and (3) time intervals of thought operation actions observed instantaneously, such as mouse button clicks. By reading the log file of Eye-Sizhi, the system extracts the time interval data for each action from the raw data and assigns lower-level interpretation results to each time segment (e.g., “*Understanding*_*Thought.all*” to the intervals of gazing at each thinking area, “*Correction*_*Statement.all*” to the intervals of typing out actions in statements, etc. in case of correction strategy). This corresponds to processing from raw data to lower-level interpretation as in Fig. 4.

The applied results of the lower-level interpretation rules are displayed on the timeline visualization screen (Fig. 6, lower right). The contents of this screen can be enlarged by selecting a specific time section with the mouse, making it possible to check details. In the example shown in Fig. 6, the section “1:00–2:00” has been selected from the display for the whole session (correction activities were performed for about 42 min in this case) and is enlarged to display a detailed confirmation of the gaze segment information for the target statement. Mouse-over causes Eye-Sizhi statement information on target gaze behaviors (statement ID: *36*, Sizhi-tag: “*result* (*結果* in Japanese),” grounds statement ID: *35*, gaze interval time) to be displayed on the tool tip screen.

2. Overall result display area: This area comprehensively displays statistical data of gaze behaviors and thought operation actions that occurred throughout the session, including relating gaze time to each thought area and key input operation, thus facilitating an understanding of to what the Eye-Sizhi user is paying attention and for how long.
3. Higher-level interpretation processing area: This area is used to display interpretation results that have been raised to the metacognition level by applying higher-level interpretation rules to lower-level interpretation results from (1).

In setting the higher-level interpretation rules, the analyst creates a new interpretation rule name in the text area of (a) and can use the combo-boxes to set and select the conditional part of the function described in “Notation for interpretation rule definition” section, as well as the label for the lower-level/higher-level interpretation results that will become the argument. Here, as a function relying on Eye-Sizhi, when the target argument is a statement, the Sizhi-tag can be specified to identify the statement. If necessary, the analyst also specifies the type of Sizhi-tag of the evidence statements and statement ID as optional settings. It is also possible to confirm and add interpretation rules in area (c); interpretation rules added in this way will be displayed in the interpretation rule display area of (d). A list that includes the lower-level interpretation rules applied in (1) is displayed in this area and can be used as an argument of the function established in (b). Pushing the “apply” button after setting a series of interpretation rules divides each applied interpretation result and displays it on the timeline visualization screen (Fig. 8 (detailed in “Applied results of interpretation rules” section)). Detailed information on the results is shown in (e).

In this way, by handling the data series measured by Eye-Sizhi comprised of gaze behaviors and thought operation actions as the semi-metacognitive monitoring and control activities to dissolve belief conflict, the interpretation process is realized. This makes it possible to visually grasp the accumulation of lower- to higher-level interpretations.

## Interpretation of thinking processes based on interpretation rules

To confirm the continuity from embodying the interpretation model to capture meta-level thinking processes shown in Fig. 1 in the interpretation framework listed in Table 1 to developing the thinking externalization application and thinking analysis support system on the basis of the framework, we demonstrate an example of the operation of the developed systems.

### Target data

Using the Eye-Sizhi described in “Eye-Sizhi” section, correction data on thoughts to dissolve belief conflicts were collected (corresponding to Table 1 measurement phase (D)). Specifically, we used a meta-level thinking method education program for nursing (Kanou et al. 2013) as an opportunity to present belief conflict dissolution thinking in nursing as a theme, and ran an actual implementation of Eye-Sizhi on their thinking results (case) as recorded by Sizhi. In the Nursing Thinking Method Training Program, which has been running since 2012, Eye-Sizhi has been implemented since the end of 2015. Currently, there are 26 cases of correction data for 2 correctors working on 14 nurses’ results.

Because one of the important thinking skills is to understand belief conflict as an opportunity for knowledge creation, in this educational program, correctors evaluated whether or not conflicts had arisen from the formation of a logical, rational structure. In addition, because metacognitive monitoring and control were performed under these conditions with the objective of correcting nurses’ thinking results, we expect one part of this metacognition will be exposed as semi-metacognitive monitoring and control in the form of gaze behaviors and corrective actions (thought operation actions). Furthermore, during corrections, we did not set the program end time as usual; instead, we allowed corrections until correctors were satisfied.

### Applied results of interpretation rules

**Fig. 7:**
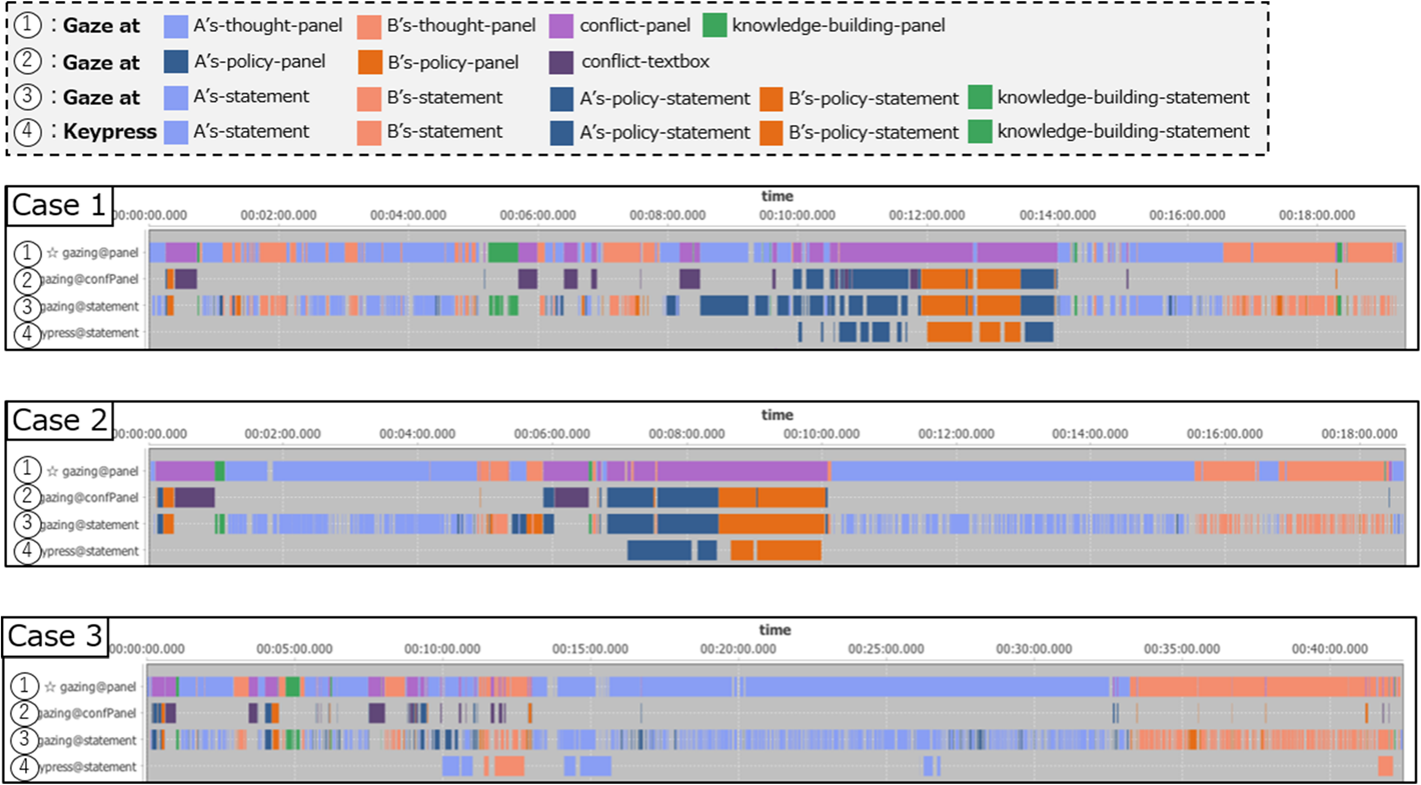
Result examples of applied lower-level interpretation rules

The applied results of the lower-level interpretation rules (part of the timeline visualization screen) for the case correction data are shown in Fig. 7 as an example of the analysis phase (E) in Table 1. In this example, we apply the four lower-level interpretation rules listed in Table 3 on the basis of the correspondence between the thinking representation objects for thought externalization and the output concepts of base-level thinking activities defined in the ontology. Corresponding to each interpretation rule, the following lower-level interpretation results of the corrector’s actions are displayed in the timeline: (1) understanding four thinking areas (*lr*_*1*_) by gaze actions; (2) understand thought contents of the conflict area by gaze actions (*lr*_*2*_: policy statement of A’s-thought, policy statement of B’s-thought, conflict text); (3) understand the statement contents (*lr*_*3*_) by gaze actions; and (4) edit actions to the statement text using the keyboard (*lr*_*4*_).

**Table 3:** Lower-level interpretation rules

*lr*_*1*_ (*GazeAt*(*Thinking*_*Area*[*all*]), “*Understanding*_*Thought.all*”)	
*lr*_*2*_ (*GazeAt*(*Conflict*_*Area*[*all*]), “*Understanding*_*Conflict.all*”)	
*lr*_*3*_ (*GazeAt*(*Statement*[*all]*), “*Understanding*_*Statement.all*”)	
*lr*_*4*_ (*Keypress*(*Statement*[*all*]), “*Correction*_*Statement.all*”)	

The timeline visualization results of (1) and (2) show that the correctors do not make chaotic gaze movements but spend their time in each thought area to grasp and correct thoughts described by the nurses. In addition, in the three cases shown here, one can see that immediately after starting the correction, the corrector moves from understanding the content described in the conflict area (purple) to beginning to dig down into the conflict in order to grasp its logical structure.

**Fig. 8:**
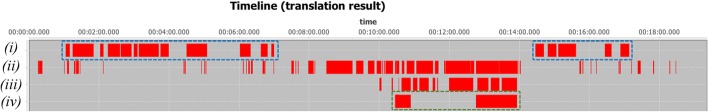
Result examples of applied higher-level interpretation rules

Figure 8 shows the results of applying higher-level interpretation rules. This is where the four types of higher-level interpretation rules listed in Table 4 are applied to case 1 in Fig. 7.

**Table 4:** Higher-level interpretation rules

*hr*_*1*_ ((*BEFORE* (*lr*_*1*_*.A*’*s*-*thought*, *lr*_*1*_*.B*’*s*-*thought*, *5000*) *OR BEFORE* (*lr*_*1*_*.B*’*s*-*thoughtB*, *lr*_*1*_.*A*’*s*-*thought*, *5000*)), “*Compare*_*A*’*s*-*Thought*_*and*_*B*’*s*-*thought*”)	
*hr*_*2*_(*ALL* (*lr*_*3*_*.policy*), “*Understanding*_*Statement.policy*”)	
*hr*_*3*_ (*ALL* (*lr*_*4*_*.policy*), “*Correction*_*Statement.policy*”)	
*hr*_*4*_ (*BEFORE* (*hr*_*2*_, *hr*_*3*_, *5000*), “*Consideration*_*Policy*_*leading*_*to*_*Conflict*”)	

In Fig. 8, the application results of each interpretation rule are classified and visualized. Row (i) represents a section in which the corrector compares A’s-thought and B’s-thought due to the application of higher-level interpretation rule *hr*_*1*_. More specifically, in the conditions of *hr*_*1*_, sections of thinking activity about A’s-thought and B’s-thought, which are consecutive within 5000 ms are extracted from the application results of lower-level interpretation rule *lr*_*1*_, and it is interpreted that the two thoughts, which are sources of conflict (A’s-thought, B’s-thought), are compared (metacognitive monitoring) by a corrector (*Compare*_*A*’*s*-*Thought*_*and*_*B*’*s*-*thought*). These results indicate that this corrector performed metacognitive monitoring to compare A’s-thought and B’s-thought in the first and second halves of the correction activity (Fig. 8, in the blue frame). Higher-level interpretation rules *hr*_*2*_ and *hr*_*3*_ extract a section of the understanding process of a policy statement that forms a belief conflict (*hr*_*2*_) and a correction section of a policy statement (*hr*_*3*_), respectively.

Furthermore, higher-level interpretation rule *hr*_*4*_ extracts the metacognitive thinking process section by designating the interpretation result of *hr*_*2*_ and *hr*_*3*_ as the condition. The application result is shown in Fig. 8(iv). Here, a section in which the corrector exerted *metacognitive monitoring* (examining how to correct a nurse’s logically inadequate conflict) and performed *metacognitive control* (modifying the conflict) *about base*-*level thinking* (contents of two conflicting policy statements) is extracted (Fig. 8, in the green frame). Note that in this example case, the analysts (the authors) intended to extract sections as candidates of meta-level thinking by setting *hr*_*1*_ and *hr*_*4*_, and then the analysts (with a thinker in some cases) ultimately judge if the sections were valid.

Therefore, by constructing a thinking externalization application on the basis of the base-level thinking representation ontology for a given thinking task, interpretation rules can be applied to gaze behaviors and thought operation actions on thinking representation objects. This supports the possibility that one part of the metacognitive thinking process can be grasped from this example. Although the validity of the extracted interpretation section is dependent on the interpretation rules defined by the analyst, the analyst clarifies the premises for analysis to target highly implicit, latent thoughts and sets interpretation rules to enhance agreement about the types of metacognitive thinking processes from the types of semi-metacognitive monitoring and control activities under consideration. We believe our framework-based thinking analysis will contribute to the formation and accumulation of knowledge for thinking analysis methods.

## Conclusion

In this study, we proposed a framework to analyze metacognitive thinking that presumes an isomorphism between metacognitive monitoring and control for base-level thinking inside the head. We also proposed gaze behaviors and thought operation actions on the output of base-level thinking activity externalized thinking representation objects as one methodology to grasp a part of the metacognitive thinking process that cannot be observed externally. We presented the framework premise by introducing output concepts of base-level thinking activities and concepts of representation objects for thought externalization to maintain the continuity between the concept of this study and the support system for measuring and analyzing the metacognition process, as well as to enhance agreement on the design principle of the framework. Furthermore, this framework was developed from the viewpoint of structural concepts and the working processes of stakeholders: analysts, developers, and thinkers. Moreover, as a case example of system development based on this framework, we built a thinking externalization application and thinking analysis support system around the theme of a thinking task to dissolve belief conflict.

To share and compare knowledge from the analysis results and to obtain consensus among researchers about the premises of system development as well as the way to capture the target thinking (metacognition), discussion must take place on a common foundation, facilitating the generation of sound research results within a shared paradigm. There still remain many points awaiting future verification regarding the validity of the notation for interpretation rule in this framework, as well as the applicability of the interpretation results. In addition, the validity, reliability, and also the limitation of the “framework itself” need to be carefully verified over time through adapting the framework to a number of practical cases. However, using our framework, we were able to realize a thinking externalization application and thinking analysis support system, founded on a base-level thinking representation ontology. In addition, we can confirm the occurrence of the desired behavior, demonstrating the postulated possibility of capturing the thought process on the basis of the defined interpretation rules. Consequently, we believe that this research contributes to the feasibility of meeting the challenge of grasping the metacognition process and accumulating knowledge about it.

In future work, we intend to further analyze correction data obtained in the meta-level thinking method training program for nursing in “Target data” section to refine our research framework. To approach this, we have investigated processes for correcting the nurses’ outcome by correctors with abundant experience and correctors with relatively little experience in terms of the type of verbalized thoughts (Sizhi-tag) on which the trainers tend to focus and adjust these thoughts to expose the root of conflict using the clues in gazing data. In addition, we have conducted experiments using Eye-Sizhi in thinking practical lessons for first-year university students (Seta et al. 2013) to analyze the transformation of their metacognitive thinking skills. Currently, we have gathered Eye-Sizhi data of 23 students who have attended/not attended practical lessons in a situation in which each learner tries to understand and examine the predefined thought of dissolving belief conflict as a learning material. In the experiment, we have also measured learners’ traits related to logical thinking skills using inventories for evaluating critical thinking disposition (Hirayama and Kusumi 2004) and metacognitive awareness (Schraw and Dennison 1994). By analyzing the recorded Eye-Sizhi data (process of gaze behaviors) and the learners’ traits, we plan to verify the validity of this study’s hypothesis: a part of the thinking process can be grasped from gaze and thought operation actions. We also plan to evaluate the reliability of the educational effect of this program on the basis of the proposed framework.
